# Research into psychiatry trainees views around the impact COVID-19 has had on the provision of electroconvulsive therapy training

**DOI:** 10.1192/bjo.2021.448

**Published:** 2021-06-18

**Authors:** Cara Webb

**Affiliations:** Pennine Care NHS Trust

## Abstract

**Aims:**

The current COVID-19 outbreak has changed the way electroconvulsive therapy (ECT) is provided. In many areas it has been moved from the more traditional ECT suites to general surgical theatres for a number of reasons, most notably being the need to ensure adequate ventilation. The introduction of the need for PPE to be worn throughout ECT and for general hospital operating procedures to be adhered to has also been a big change. The change in the required infection control procedures has had an impact on treatment capacity and has led many areas to reduce, relocate or centralise their ECT provisions which has had a knock on effect on training.

This study assesses the perceived impact COVID-19 has had on the provision of training and learning in ECT for core and higher psychiatry trainees in the North West as well as their perceived competence levels.

**Method:**

Views were sought through surveys and focus groups from September to December 2020, participants were core and higher psychiatry trainees in the North West. Participants were recruited via email, the total population size was 87, 21 Core trainees responded and 14 higher trainees. From the survey respondents, 5 participants agreed to attend a focus group.

**Result:**

Results show that the provision of ECT training has been poor during the COVID-19 outbreak. Almost 81% of core trainees surveyed and 92.86% of higher trainees had participated in no ECT sessions from the start of the COVID-19 outbreak to the time of data collection eight to ten months later.

81% of core trainees and 85.71% of higher trainees had received no teaching in any form about ECT over the period studied.

When considering the competencies required in ECT for a core trainee, one (4.76%) felt they were fully competent, 3 (14.29%) nearly competent, 7(33.33%) needs some improvement, 10 (47.62%) not yet competent. Only one higher trainee felt they met the RCPsych competencies, 5 (35.71%) were nearly competent, 6 (42.86%) need some improvement and 2 (14.29%) were not yet competent.

**Conclusion:**

This study indicates a clear lack of provision of training which is very concerning ,and possibly pre dates the COVID outbreak, particularly in the case of specialty trainees who may well be consultants in a number of months and will not have the time to make up for the lost training. In order for ECT provision to continue it is crucial that we are able to adequately train the future workforce.

